# Pediatric Opioid‑Use‑Associated Neurotoxicity with Cerebellar Edema: A Case of POUNCE Syndrome Following Vein of Galen Aneurysm Treatment

**DOI:** 10.5334/jbsr.3786

**Published:** 2024-11-14

**Authors:** Maxime Goldfinger

**Affiliations:** 1 Chu brugmann, Belgium

**Keywords:** POUNCE syndrome, pediatric neurotoxicity, cerebellar edema, opioid‑induced encephalopathy

## Abstract

We report the case of a 4‑year‑old child who experienced rapid neurological decline following opioid administration during anesthesia for an interventional procedure to treat a vein of Galen aneurysm. Cerebral magnetic resonance imaging (MRI) revealed marked cytotoxic edema in both cerebellar hemispheres and the brainstem, indicative of opioid‑induced neurotoxicity. A follow‑up MRI, performed 2 weeks later, showed profound cerebellar and brainstem atrophy and showed reduction in mass effect due to cytotoxic edema.

*Teaching point:* Pediatric opioid‑use‑associated neurotoxicity with cerebellar edema (POUNCE) syndrome is a rare condition, characterized by cerebellar edema as a hallmark feature, which can be identified on MRI in pediatric patients following opioid use.

## Introduction

Pediatric opioid‑use‑associated neurotoxicity with cerebellar edema (POUNCE) syndrome is a rare and severe form of neurotoxicity caused by opioid overdose, primarily affecting children. It is characterized by a distinct pattern of cerebellar edema, often accompanied by varying degrees of supratentorial involvement. First recognized as a potential complication of opioid use, POUNCE syndrome is now known for its rapid onset of neurological decline following opioid administration.

We report the case of a child who, following interventional surgery for a vein of Galen aneurysm and opioid use during anesthesia, rapidly developed significant clinical deterioration. Magnetic resonance imaging (MRI) revealed cytotoxic edema involving both cerebellar hemispheres and the brainstem.

## Case Report

**Figure 1:**
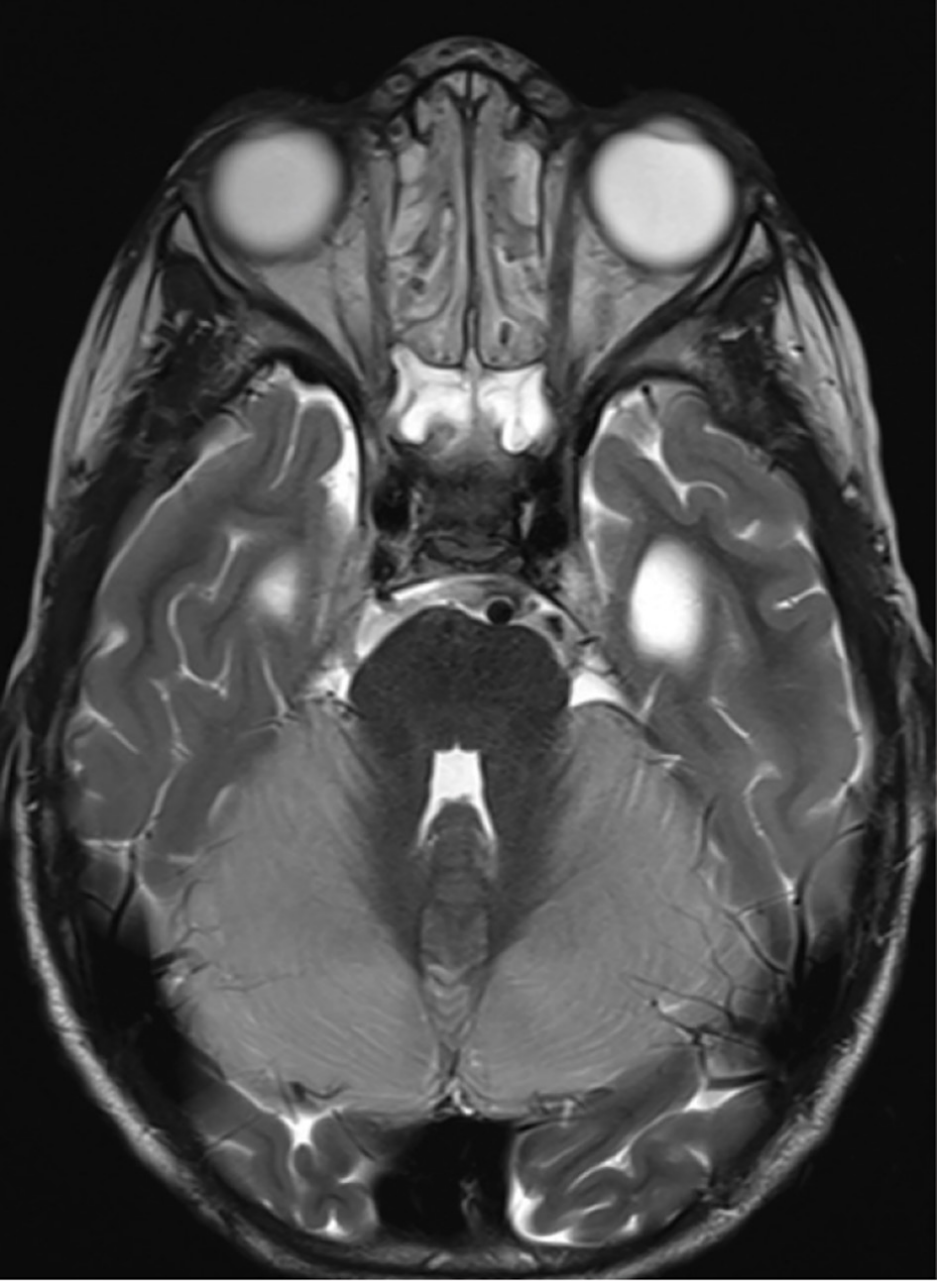
Axial T2‑weighted MRI shows a hyperintense signal in both cerebellar hemispheres and the brainstem, indicative of edema.

**Figure 2:**
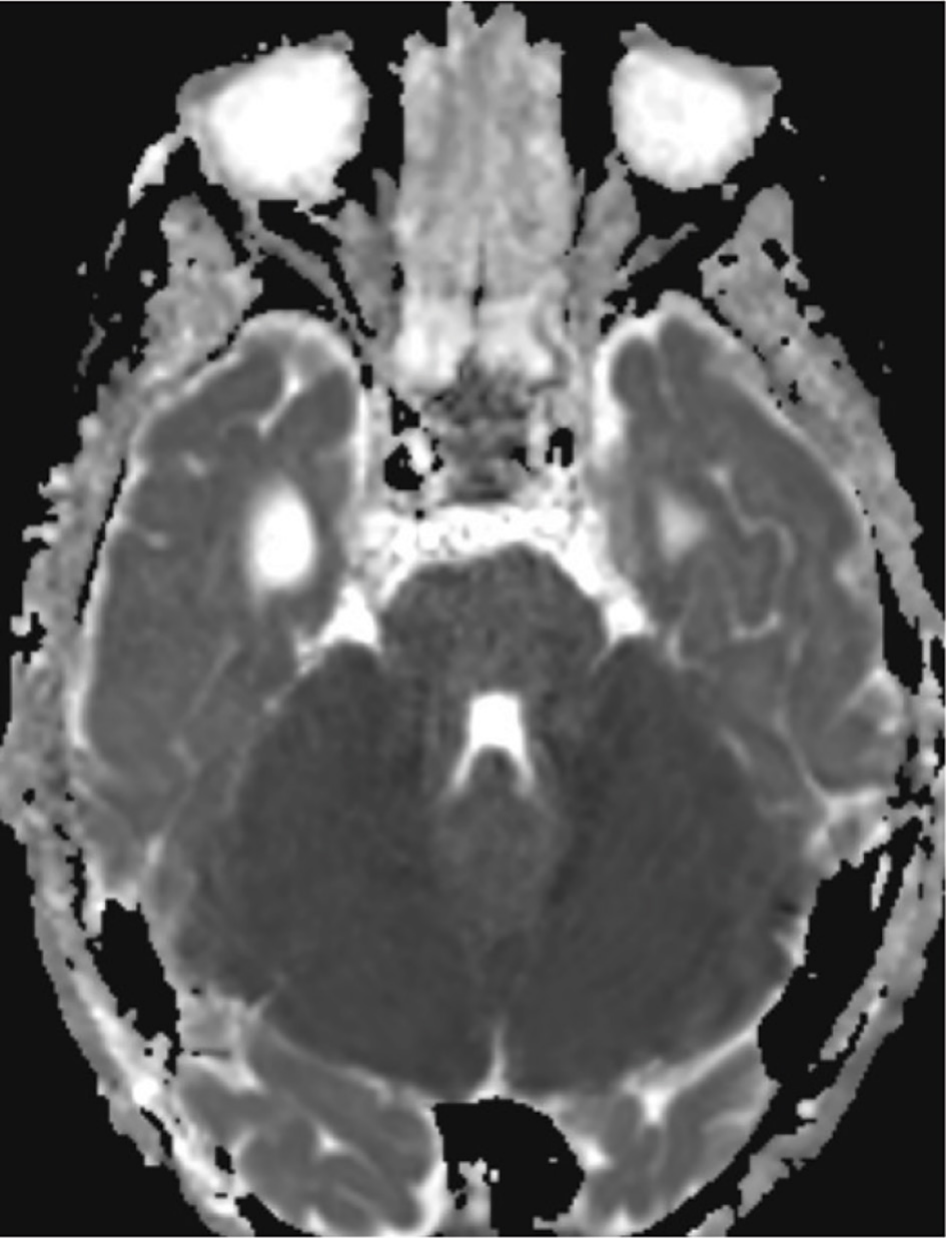
Axial ADC map demonstrates diffusion restriction predominantly in the cerebellar hemispheres and involvement of the brainstem, consistent with cytotoxic edema.

A 4‑year‑old boy was admitted following rapid neurological deterioration after undergoing interventional embolization of a vein of Galen malformation. Immediately postoperatively, the child experienced progressively altered consciousness; the Glasgow Coma Scale (GCS) score remained between 6 and 8 despite the antagonization of sufentanyl by naloxone. MRI performed after the procedure revealed a hyperintense signal in both cerebellar hemispheres on axial T2‑weighted (Figure 1) MRI and diffusion restriction predominantly in the cerebellar hemispheres and involvement of the brainstem, consistent with cytotoxic edema on the axial apparent diffusion coefficient (ADC) map (Figure 2).

**Figure 3:**
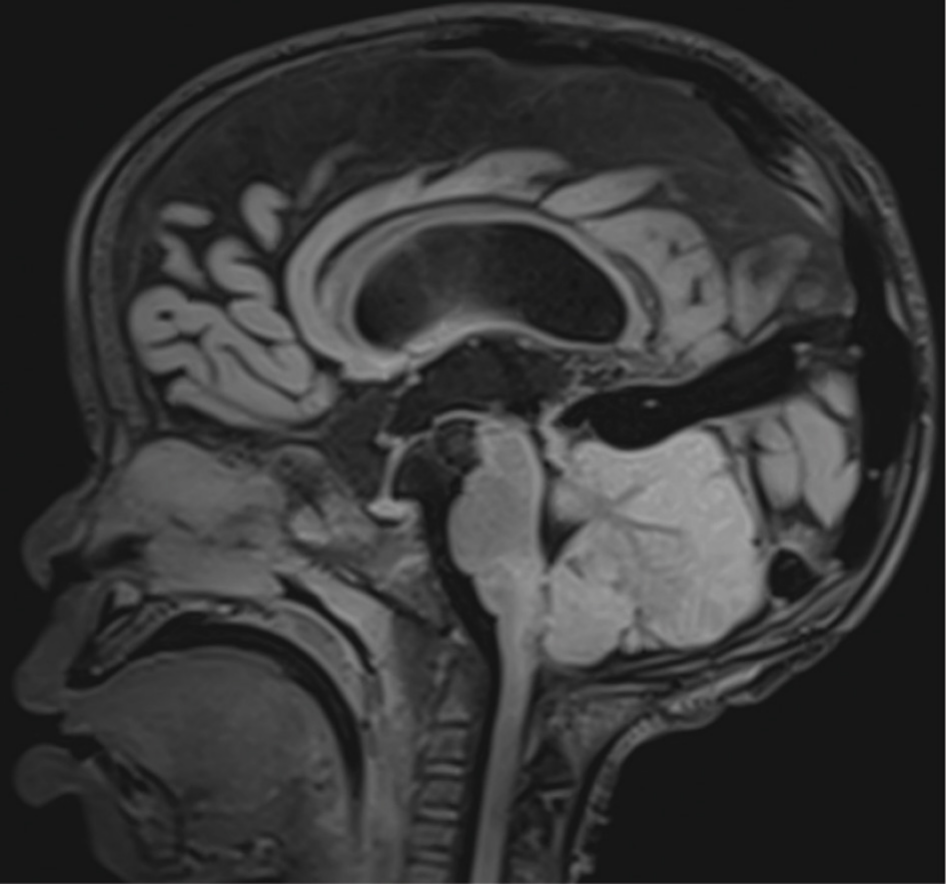
Sagittal T2‑weighted MRI showing an intense signal in the cerebellum, causing mass effect with anterior displacement of the medullar bulb.

The sagittal fluid attenuated inversion recovery (FLAIR) sequence reveals a significant mass effect due to cytotoxic edema, leading to compression of the fourth ventricle and exerting a mass effect on the medulla. However, there is no tonsillar herniation (Figure 3).

The patient benefited from supportive care, which included respiratory support, infection management, antihypertensive therapy, and specialized neurological care. Enriched enteral feeding was provided due to impaired swallowing and weight loss.

**Figure 4:**
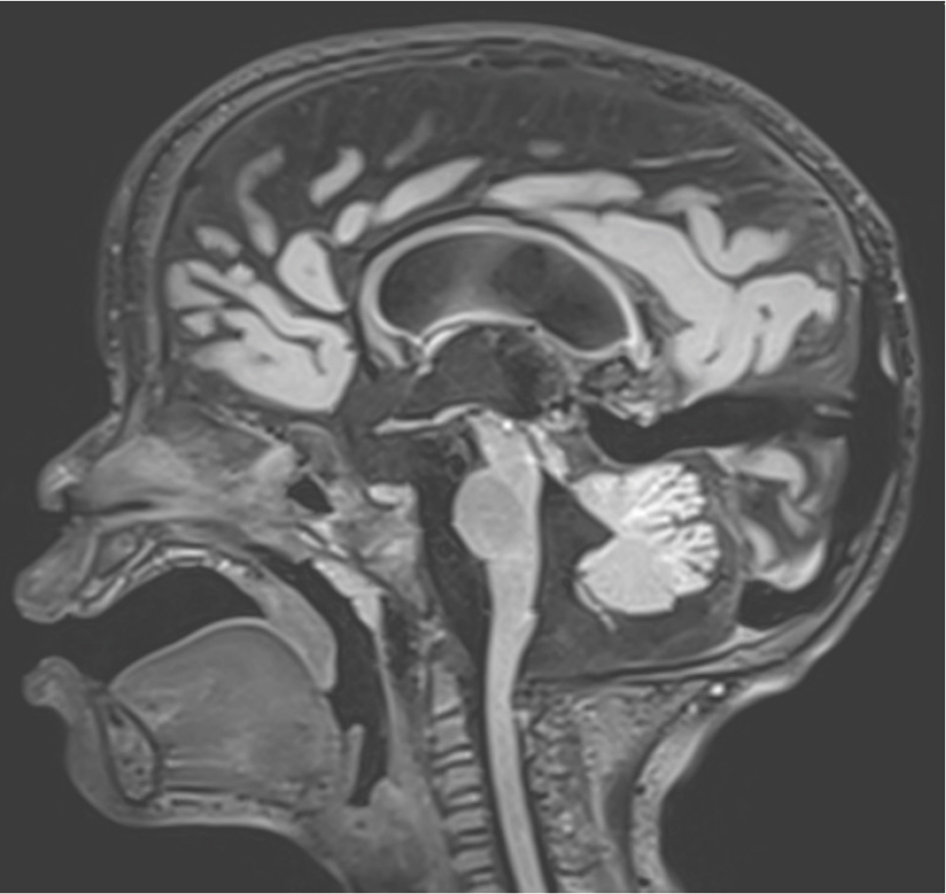
Sagittal FLAIR‑weighted MRI image showing cerebellar atrophy following opioid‑induced toxicity. A pre‑existing cystic malformation of the posterior fossa, consistent with a Blake’s pouch cyst, remains visible.

Follow‑up imaging conducted 2 weeks later showed a reduction in mass effect and cytotoxic edema, along with evidence of atrophy in the cerebellar gray matter, particularly involving the vermis and the medial cerebellar lobes. A pre‑existing cystic malformation of the posterior fossa, consistent with a Blake’s pouch cyst, remains visible (Figure 4).

On the basis of the clinical and imaging findings, along with the temporal relationship with opioid administration during anesthesia, pediatric opioid‑use‑associated neurotoxicity with cerebellar edema (POUNCE) syndrome was considered the most likely diagnosis.

After 2 months, the patient’s progress has been very positive, with significant neurological recovery. Despite some persistent sequelae, such as mild left hemiparesis and slight spasticity in the left plantar flexors, the patient is now able to walk with anterior support and demonstrates preserved fine motor skills.

## Discussion

The clinical history of our patient aligns with previously reported cases of POUNCE syndrome [1–3]. Although rare, this syndrome is now recognized as a potentially serious complication of opioid use in children, either accidentally or as a result of opioid use during anesthesia [1–3]. The rapid onset of neurological symptoms after opioid administration, coupled with imaging findings of cerebellar cytotoxic edema, closely matches the syndrome’s characteristic presentation.

Early recognition is crucial, as it allows for timely therapeutic management, including opioid cessation, supportive care, and, when needed, surgical intervention [1–2]. Thanks to prompt diagnosis and treatment, significant neurological recovery is possible, and many patients can regain important functions. While some sequelae may persist, the potential for neurological improvement through neuroplasticity is substantial, especially with early intervention and continued rehabilitation, which can greatly enhance the patient’s long‑term quality of life [3].

This case underscores the importance of early diagnosis and awareness of POUNCE syndrome in pediatric patients, particularly following opioid administration, to minimize long‑term neurological damage.

## Conclusion

Pediatric opioid‑use‑associated neurotoxicity with cerebellar edema (POUNCE) syndrome is characterized by distinctive imaging findings, particularly cerebellar and brainstem edema, which are crucial for diagnosis. MRI plays a central role in identifying the hallmark features of this syndrome, enabling early diagnosis and timely intervention. Radiologists should be vigilant in recognizing these patterns to facilitate prompt management and improve patient outcomes.
